# Chromosomal Damage, Chromosome Instability, and Polymorphisms in *GSTP1* and *XRCC1* as Biomarkers of Effect and Susceptibility in Farmers Exposed to Pesticides

**DOI:** 10.3390/ijms25084167

**Published:** 2024-04-10

**Authors:** Fernando Aldana-Salazar, Nelson Rangel, María José Rodríguez, César Baracaldo, María Martínez-Agüero, Milena Rondón-Lagos

**Affiliations:** 1 School of Biological Sciences, Universidad Pedagógica y Tecnológica de Colombia, Tunja 150003, Colombia; luis.aldana02@uptc.edu.co (F.A.-S.); maria.rodriguez20@uptc.edu.co (M.J.R.); 2 Departamento de Nutrición y Bioquímica, Facultad de Ciencias, Pontificia Universidad Javeriana, Bogotá 110231, Colombia; 3 Doctoral Program in Biological and Environmental Sciences, Universidad Pedagógica y Tecnológica de Colombia, Tunja 150003, Colombia; cesar.baracaldo@uptc.edu.co; 4 Centro de Investigaciones en Microbiología y Biotecnología-UR (CIMBIUR), Facultad de Ciencias Naturales, Universidad del Rosario, Bogotá 110231, Colombia; maria.martinez@urosario.edu.co

**Keywords:** pesticides, chromosomal instability, clonal heterogeneity, polymorphism, FISH, restriction enzymes

## Abstract

In the department of Boyacá, Colombia, agriculture stands as one of the primary economic activities. However, the escalating utilization of pesticides within this sector has sparked concern regarding its potential correlation with elevated risks of genotoxicity, chromosomal alterations, and carcinogenesis. Furthermore, pesticides have been associated with a broad spectrum of genetic polymorphisms that impact pivotal genes involved in pesticide metabolism and DNA repair, among other processes. Nonetheless, our understanding of the genotoxic effects of pesticides on the chromosomes (as biomarkers of effect) in exposed farmers and the impact of genetic polymorphisms (as susceptibility biomarkers) on the increased risk of chromosomal damage is still limited. The aim of our study was to evaluate chromosomal alterations, chromosomal instability, and clonal heterogeneity, as well as the presence of polymorphic variants in the *GSTP1* and *XRCC1* genes, in peripheral blood samples of farmers occupationally exposed to pesticides in Aquitania, Colombia, and in an unexposed control group. Our results showed statistically significant differences in the frequency of numerical chromosomal alterations, chromosomal instability, and clonal heterogeneity levels between the exposed and unexposed groups. In addition, we also found a higher frequency of chromosomal instability and clonal heterogeneity in exposed individuals carrying the heterozygous *GSTP1* AG and *XRCC1* (exon 10) GA genotypes. The evaluation of chromosomal alterations and chromosomal instability resulting from pesticide exposure, combined with the identification of polymorphic variants in the *GSTP1* and *XRCC1* genes, and further research involving a larger group of individuals exposed to pesticides could enable the identification of effect and susceptibility biomarkers. Such markers could prove valuable for monitoring individuals occupationally exposed to pesticides.

## 1. Introduction

The extensive use of pesticides and the prolonged and consistent exposure of agricultural workers to these chemicals render the evaluation of carcinogenic and mutagenic risks a significant public health concern. Despite the fact that for some years, several investigations have focused on the evaluation of the cytotoxic and genotoxic damage induced by exposure to pesticides, there are very few studies that have inquired into the chromosomal damage caused in farmers exposed to them. Chromosomal damage related to pesticide exposure has been considered as a biomarker of effect, and despite studies reporting notable differences in the frequency of chromosome alterations (CAs) between exposed individuals and unexposed controls [1,2,3,4,5,6], others have not observed any association [7,8]. However, it is important to highlight that in these studies, the evaluation of chromosomal damage has been conducted through methods such as sister chromatid exchange (SCE) [9] and micronuclei (MN), among others. Therefore, studies that report the type and frequency of CAs, as well as the level of chromosomal instability (CIN) caused by exposure to pesticides, are scarce. Additionally, it has been suggested that while oxidative stress, epigenetic modifications, and gut microbiota modulation are recognized as key pathogenic mechanisms induced by pesticide exposure, their biological impacts appear to be primarily influenced by the presence of genetic polymorphisms. In fact, it has been demonstrated that pesticides are linked to a broad spectrum of genetic polymorphisms affecting crucial genes involved in the regulation of the cell cycle, redox status, drug metabolism, pesticide metabolism, and DNA repair [10]. Moreover, the toxic response resulting from pesticide exposure may vary among organisms, influenced by genetic and physiological factors such as age, diet, nutritional status, hormonal balance, and overall health. Indeed, it has been suggested that certain individuals may be more susceptible to oxidative stress induced by pesticides, thereby increasing their health risks owing to the influence of genetic polymorphisms [10]. Interindividual differences can be evaluated through susceptibility biomarkers. These can include polymorphic variants in genes encoding enzymes involved in xenobiotic metabolism (such as Glutathione S transferase P1 gene—*GSTP1*) and DNA break repair (such as X-ray repair cross complementing group 1 gene—*XRCC1*). Genetic variations in these genes may influence the genotoxicity resulting from pesticide exposure [11]. For example, the *GSTP1* gene has been associated with glutathione conjugation detoxification catalyzed by glutathione transferases. In cases where this detoxification process is ineffective, the toxic by-products can cause damage to intracellular molecules [12]. Of note, several GST enzymes expressed in the liver are polymorphic [13] and may impact pesticide metabolism and detoxification [14], potentially affecting normal cell function. Moreover, it has been shown that the enzymatic activity of GSTP1 is highly influenced by a single-nucleotide polymorphism known as the A313G polymorphism. This polymorphism results in the substitution of isoleucine (Ile) with valine (Val) at the 105 amino acid position, referred to as Ile105Val, thereby generating three GSTP1 genotypes: Ile/Ile homozygous wild type, Ile/Val heterozygous variant, and Val/Val homozygous variant [15]. The Ile105Val polymorphism of *GSTP1* has been associated with decreased enzyme metabolic activity [14], leading to the accumulation of toxic metabolites in the body [16]. *XRCC1* is a single- and double-strand DNA break repair gene that plays an important role in protection against pesticide-related genotoxicity [11]. In fact, polymorphisms at codon 194 in exon 6 (C→T) and at codon 399 in exon 10 (G→A) in the *XRCC1* gene have been utilized as biomarkers to assess susceptibility to the DNA damage induced by pesticides [17,18]. 

This, along with the deficiency of studies carried out in our country on the genotoxic damage that exposure to pesticides can cause, highlights the need to deepen and expand our knowledge on the level of CIN caused by exposure to pesticides. Additionally, there is a need to investigate the presence of polymorphic variants in the *GSTP1* and *XRCC1* genes. This could confer susceptibility to the development of diseases resulting from exposure to pesticides. This study aimed to determine the type and frequency of chromosomal alterations, evaluate the levels of CIN and CH, and establish their associations with polymorphic variants at exon 5 (A→G) in the *GSTP1* gene and at exons 6 (C→T) and 10 (G→A) in the *XRCC1* gene in a group of ten farmers who had been occupationally exposed to pesticides in the town of Aquitania, Colombia, and in ten unexposed individuals. GTG banding, fluorescence in situ hybridization (FISH), and restriction fragment length polymorphisms (RFLP) were used. 

Our results showed statistically significant differences in the frequency of numerical chromosomal alterations (NCAs), CIN, and CH levels between the exposed and unexposed groups. Furthermore, the exposed group carrying the heterozygous genotypes *GSTP1* AG and *XRCC1* (exon 10) GA exhibited a higher frequency of NCAs, CIN, and CH compared to the unexposed group. These results suggest that individuals exposed to pesticides and carrying the *GSTP1* AG or *XRCC1* GA genotypes may be more susceptible to an elevated risk of DNA damage.

## 2. Results

### 2.1. Study Groups

The exposed group comprised both male and female individuals aged between 19 and 71 years. All had been engaged in pesticide spraying/handling and exposed to pesticides through work for a minimum of 12 months (Table 1 and Supplementary Table S1). The average duration of pesticide exposure was 180.2 months, and the frequency of pesticide exposure was primarily once a week or twice a month (Table 1 and Table 2). 

The routes of exposure were mainly dermal and respiratory (Table 2). Minor routes of exposure to pesticides, such as parenteral exposure (intramuscular, subcutaneous or intravenous), unintentional (accidental) oral exposure, and/or eye/ear exposure, were not reported by the exposed group. The methods used by the exposed group for the irrigation of pesticides were machines, pumps, and mixed (machines and pumps), with an irrigation frequency that oscillated between once a week and twice a month (Table 2). The exposed group reported that during the fumigation and/or pesticide handling process, they used minimal protection measures including gloves and masks.

The unexposed group comprised 10 healthy individuals, both male and female, who had no history of occupational exposure to pesticides. Similar to the exposed group, the unexposed group spanned an age range of 19 to 71 years and exhibited comparable gender distribution and lifestyle habits (Table 1). Results are presented as the mean ± standard deviation (SD) (Table 2). The exposed and unexposed groups indicated a low incidence of smoking and alcohol intake. The pesticide mixtures mainly used by farmers were furadan, malathion, antracon, manzate, parathion, curacron, and fitoraz.

### 2.2. High Frequency of Chromosomal Alterations in Exposed Individuals 

Cytogenetic analysis by GTG banding demonstrated a modal diploid number (2n) in both the exposed and unexposed groups. A total of 731 metaphases were analyzed. The exposed group exhibited significantly higher frequencies of numerical chromosomal alterations (NCAs) compared to what was observed in the unexposed group (151 and 33, respectively) (*p* ≤ 0.0001 **; Fisher’s exact test) (Table 3).

While no statistically significant differences were observed between the exposed and unexposed groups in any cases, the frequency of SCAs, chtb/chrb, and fragilities fra was higher in the exposed group. The above results suggest chromosomal damage attributable to pesticide exposure (Table 3). 

Statistical analysis revealed significant differences in the frequency of NCAs, SCAs, and chrb/chtb between paired exposed and unexposed individuals (*p* ≤ 0.01 **; Fisher’s exact test) (Table 4).

**Figure 1:**
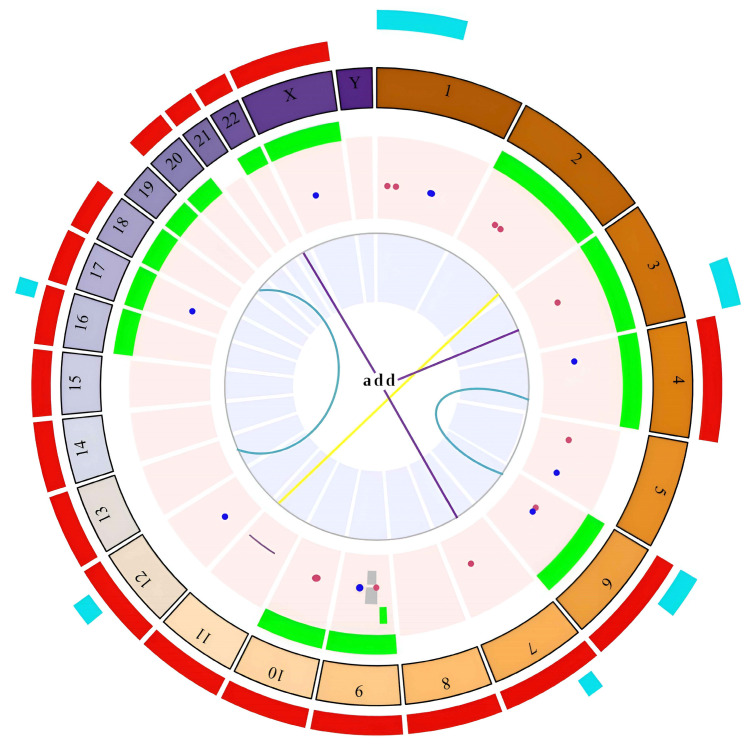
Circos plot of the chromosomal alterations observed in the exposed group. The outer blue-cyan ring indicates the presence of deletions, including del(1)(p12) and del(9)(q11). The red ring indicates losses of whole chromosomes. The next ring indicates the number of chromosomes. The green ring highlights the gain of whole chromosomes. The following ring denotes chromosomal abnormalities that impacted only one chromosome or where only one chromosome was identified. These alterations include the following: fragilities including fra(9)(q12) and fra(1)(q12), represented as dark blue dots; chromatid breaks including chtb(9)(q12) and chtb(1)(p36), represented as dark red dots; inversions including inv(9)(p12q13), represented as gray rectangles; and a derivative chromosome der(11)del(11)(p12)del(11)(q23), represented as a dark brown line. The last ring in the center of the circos diagram represents chromosomal alterations involving at least two chromosomes: blue arcs indicate translocations including t(4;6)(q31;p25) and t(13;19)(p13;p13); purple lines are additional material of unknown origin including add(3)(q29) and add(22)(q13); and the yellow line represents a derivative chromosome including der(2)t(2;11)(q37.3;q31). The circos diagram was created using the R statistical software (version 4.3.1) with the BioCircos library, and it was subsequently edited in PowerPoint (version 2180) to add some symbols representing alterations not found in the mentioned library.

Particularly, in the exposed group (E), the following frequencies were observed: 151 NCAs in all individuals (100%), 20 SCAs in nine individuals (90%), 20 chtb/chrb in six individuals (60%), 42 fra in eight individuals (80%), and 4 chromosomal heteromorphisms in two individuals (20%) (Figure 1 and Table 3 and Table 4). Within the NCAs, gains (trisomies, endoreduplications, and polyploidies) were observed more frequently (72%) than losses (monosomies) (28%). Chromosomes 18 (11.9%), X (9.5%), and 12 (9.5%) exhibited the highest frequencies of monosomies. Additionally, marker chromosomes were observed with a higher frequency among the gains (34.8%), followed by endoreduplications (19.26%) and polyploidies (15.6%). Regarding SCAs, a total of 20 were observed in 90% of the exposed individuals (Table 4 and Figure 1).

Among the SCAs, the most frequent were the del (30%), followed by inversions (inv) (25%) and additional material of unknown origin (add) (20%). Less frequently observed structural alterations included translocations (t) (10%), derived chromosomes (der) (10%), and duplications (dup) (5%). Additionally, a total of 20 chrb/chtb were identified in 60% of the exposed individuals (Table 3 and Table 4). The most frequent was chtb(9)(q12), observed in 50% of the exposed individuals. Moreover, 42 fragilities (fra) were found in the exposed group, and the most frequent was the fra(9)(q12) (67.4%), observed in 70% of the exposed individuals.

In the unexposed group (UE), 33 NCAs were identified in eight individuals (80%), 15 SCAs in seven individuals (70%), 7 chtb/chrb in four individuals (40%), 22 fra in seven individuals (70%), and 5 chromosomal heteromorphisms in two individuals (20%) (Table 3 and Table 4). Within the NCAs, losses (monosomies) (63.63%) were observed more frequently than were gains (trisomies, endoreduplications, and polyploidies) (36.36%). Chromosomes 5 (14.28%) and 8 (14.28%) exhibited the highest frequencies of monosomies. Among the gains, marker chromosomes were observed at a higher frequency (33.3%), followed by polyploidies (25%). A total of 15 SCAs were identified in 70% of the exposed individuals (Table 3 and Table 4). Among the SCAs, the most frequent were the del (40%) and inv (33.33%). Other SCAs observed less frequently in this group included add (13.3%). 

### 2.3. High Levels of Numerical Chromosomal Instability (CIN) in Exposed Individuals

**Figure 2:**
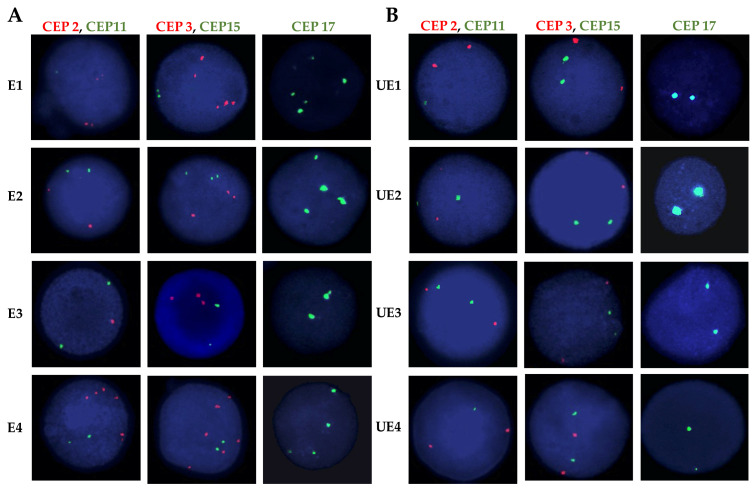
Representative FISH images of exposed (E) and unexposed individuals (UE). Interphase nuclei are shown for each case. (**A**) Exposed individuals exhibited chromosomal instability, as evidenced by the presence of more than two signals for chromosomes 2, 3, 11, 15, and 17. (**B**) In the unexposed individual, a normal number of signals (two signals) was observed for each of the indicated chromosomes above. For assessing the copy number of chromosomes 2 and 11, as well as 3 and 15, Dual-color FISH was employed, whereas single FISH was used for evaluating the copy number of chromosome 17. Centromeric probes (CEPs) were labeled with various spectrum colors: spectrum orange for CEP2 and CEP3 and spectrum green for CEP11, CEP15, and CEP17.

**Figure 3:**
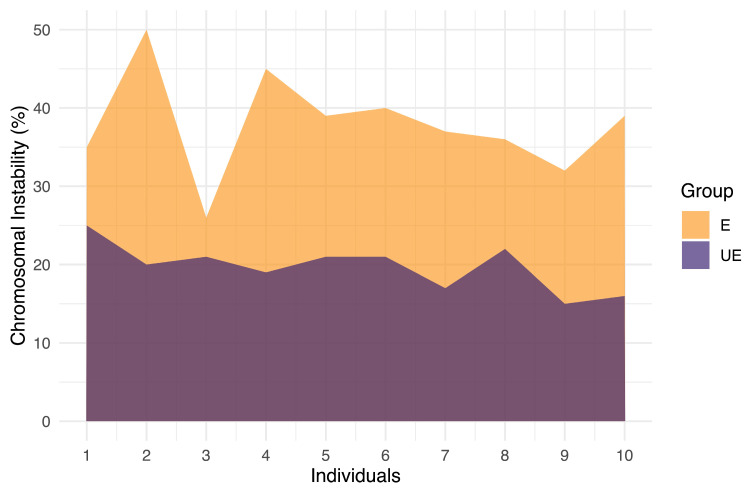
Chromosomal Instability (CIN) in the exposed and unexposed groups The exposed group was classified as having high CIN (CIN ≥ 25). The unexposed group was classified as having low CIN (CIN < 25%). In each individual, 100 interphase nuclei were analyzed.

CIN was assessed in 100 interphase nuclei using dual-color FISH assays. While all exposed individuals exhibited high levels of CIN (CIN ≥ 25%), unexposed individuals demonstrated low CIN (CIN ≤ 14%) (Figure 2 and Figure 3). Specifically, CIN ranged between 26% and 50% in the exposed group, while in the unexposed group, CIN ranged between 15% and 25% (Figure 2 and Figure 3, and Supplementary Table S2). These differences were statistically significant (*p* ≤ 2.953 × 10^−7^. Student’s *t*-test).

In the same way, chromosomes with more or less stable aneuploidy in both the exposed and unexposed groups were identified by using the Kruskal–Wallis test. No statistically significant differences were observed between the chromosomes analyzed in the exposed individuals. This could be due to the high CIN found in all chromosomes (30.2%–46.9%) (Supplementary Figure S1A). However, chromosome 3 had the highest percentage of CIN, while chromosomes 2 and 15 had the lowest percentage. Even so, they were classified as high CIN (Supplementary Figure S1A). This test showed a statistically significant difference in the unexposed group, (*p* ≤ 0.003552 **) between chromosomes 2 and 17, with chromosome 2 being the most stable (Supplementary Figure S1B).

### 2.4. High CH in Exposed Individuals and Intermediate CH in Unexposed Individuals 

**Figure 4:**
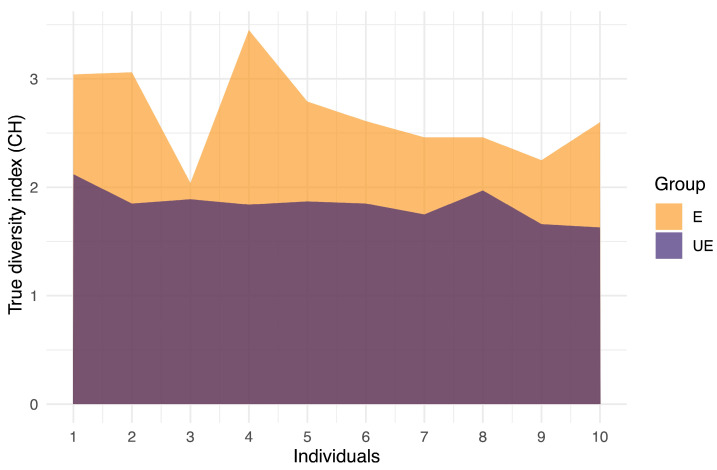
Clonal heterogeneity (CH) in the exposed and unexposed groups. CH was determined by using the true diversity index (TD). The exposed group showed high CH, while the unexposed group showed intermediate CH. According to the level of CH, each exposed and unexposed individual was classified as having low CH (<1.5), intermediate CH (CH > 1.62 < 2), or high CH (CH > 2).

The true diversity index (TD) was used to determine clonal heterogeneity (CH) in both groups. TD integrates the number and abundance of different cell populations in the same individual. Exposed individuals displayed higher CH (CH > 2) as compared to the intermediate CH (CH > 1.62 < 2) observed in unexposed individuals. In the exposed group, CH exhibited a higher level (TD = 2.67) than in the unexposed group (TD = 1.84) (Supplementary Table S2). This difference was statistically significant (*p* ≤ 0.00002165 ***; Non-parametric Mann–Whitney Wilcoxon) (Figure 4). 

Moreover, the CH was also evaluated for each of the chromosomes studied in both groups. Specifically, in the exposed group, statistically significant differences (*p* ≤ 0.00181 **) were noted between chromosomes 11 and 3 and between chromosomes 15 and 3 (with chromosome 3 being the one with the highest CH). Statistically significant differences (*p* ≤ 0.01251 **) were also noted in the unexposed group but between chromosomes 2 and 17, with chromosome 17 presenting the highest CH. 

### 2.5. Association between Variables 

**Figure 5:**
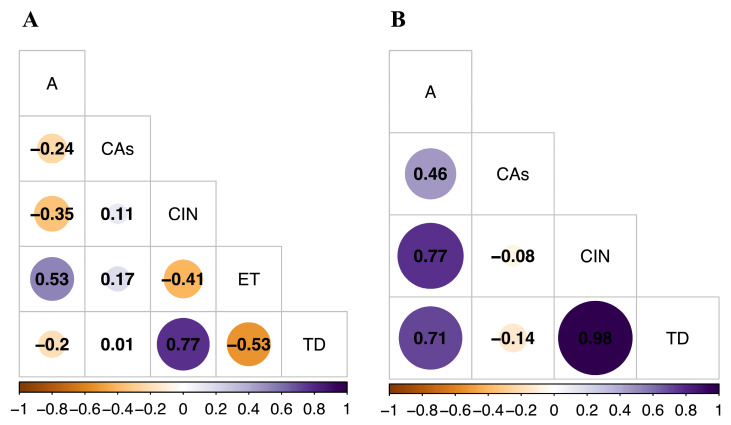
Multivariate analysis using the Spearman correlation coefficient for (**A**) the exposed group and (**B**) the unexposed group. Values greater than 0.5 indicate a statistically significant correlation. The strength of correlation is categorized as follows: very low (0–0.199), low (0.2–0.399), moderate (0.4–0.599), strong (0.6–0.799), and very strong (0.8–1).

Correlations between the frequency of chromosomal alterations (CAs), the levels of chromosomal instability (CIN) and chromosomal heterogeneity (TD), and variables such as age and pesticide exposure time (ET) were examined in the exposed and unexposed groups using Spearman correlation coefficient analysis. In the exposed group, a positive correlation was noted between CIN and TD, while a negative correlation was observed between TD and ET (Figure 5A). Meanwhile, in the unexposed group, a positive correlation was noted between age with CIN and TD (Figure 5B). Smoking and alcohol consumption variables were not evaluated due to the low reported consumption in both groups.

### 2.6. Polymorphisms in the GSTP1 and XRCC1 Genes

In order to explore whether individual genetic variations in xenobiotic metabolization and DNA damage repair might impact susceptibility to DNA damage from pesticide exposure, individuals from both groups were genotyped for the *GSTP1* and *XRCC1* genes. The distribution of *GSTP1* exon 5, *XRCC1* exon 6, and *XRCC1* exon 10 genotypes in both study groups were consistent with Hardy–Weinberg equilibrium (*p* > 0.05). Details regarding genotype distribution and variant allele frequencies in the individuals studied are provided in Table 5.

**Figure 6:**
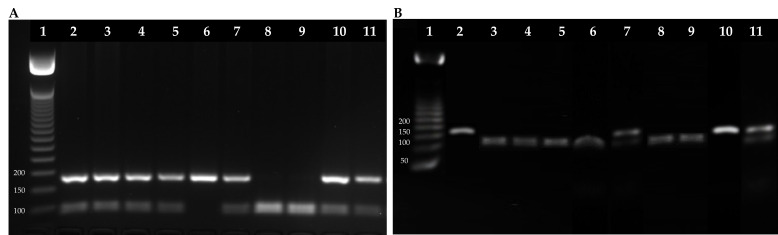
The restriction profile of *GSTP1* exon 5 for exposed and unexposed individuals. (**A**) Exposed individuals. Lane 1: DNA ladder (50–1500 bp); lane 2: exposed 1, AG genotype (heterozygous, polymorphic); lane 3: exposed 2, AG genotype; lane 4: exposed 3, AG genotype; lane 5: exposed 4, AG genotype; lane 6: exposed 5, AA genotype (homozygous, wild type); lane 7: exposed 6, AG genotype; lane 8: exposed 7, GG genotype (homozygous, polymorphic); lane 9: exposed 8, GG genotype; lane 10: exposed 9, AG genotype; lane 11: exposed 10, AG genotype. (**B**) Unexposed individuals. Lane 1: DNA ladder (50–1500 bp); lane 2: unexposed 1, AA genotype (homozygous, wild type); lane 3: unexposed 2, GG genotype (homozygous, polymorphic); lane 4: unexposed 3, GG genotype; lane 5: unexposed 4, GG genotype; lane 6: unexposed 5, GG genotype; lane 7: unexposed 6, AG genotype (heterozygous, polymorphic); lane 8: unexposed 7, GG genotype; lane 9: unexposed 8, GG genotype; lane 10: unexposed 9, AA genotype; lane 11: unexposed 10, AG genotype.

For the *GSTP1* gene at exon 5, the AG heterozygous genotype was more frequent in exposed individuals than in unexposed individuals (70% and 20%, respectively), representing a statistically significant difference (*p* < 0.0001 **). In contrast, the GG homozygous genotype was more frequent in unexposed individuals than in exposed individuals (60% and 20% respectively), and this difference was also statistically significant (*p* < 0.0001 **) (Table 5 and Figure 6). 

**Figure 7:**
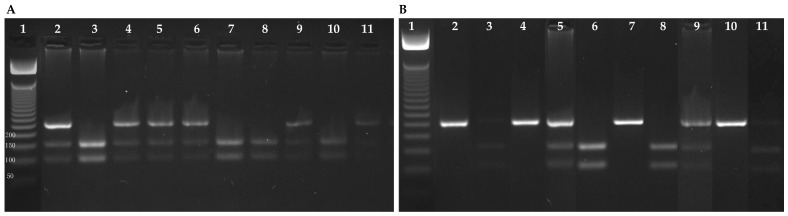
The restriction profile of *XRCC1* exon 10 for exposed and unexposed individuals. (**A**) Exposed individuals. Lane 1: DNA ladder (50–1500 bp); lane 2: exposed 1, GA genotype (heterozygous, polymorphic); lane 3: exposed 2, AA genotype (homozygous, polymorphic); lane 4: exposed 3, GA genotype; lane 5: exposed 4, GA genotype; lane 6: exposed 5, GA genotype; lane 7: exposed 6, AA genotype; lane 8: exposed 7, AA genotype; lane 9: exposed 8, GA genotype; lane 10: exposed 9, AA genotype; lane 11: exposed 10, GA genotype. (**B**) Unexposed individuals. Lane 1: DNA Ladder (50–1500 bp); lane 2: unexposed 1, GG genotype (homozygous, wild type); lane 3: unexposed 2, GA genotype (heterozygous, polymorphic); lane 4: unexposed 3, GG genotype; lane 5: unexposed 4, GA genotype; lane 6: unexposed 5, AA genotype (homozygous, polymorphic); lane 7: unexposed 6, GG genotype; lane 8: unexposed 7, AA genotype; lane 9: unexposed 8, GA genotype; lane 10: unexposed 9, GG genotype; lane 11: unexposed 10, GA genotype.

Regarding the polymorphisms at exon 6 in the *XRCC1* gene, no significant differences were observed between the genotypes of the exposed and unexposed individuals. The CC genotype was the most frequent in the two groups studied (80%) (Table 5). Contrary to what was observed at exon 6 in the *XRCC1* gene, at exon 10, the GA heterozygous genotype was more frequent in exposed individuals than in unexposed individuals (60% and 40%, respectively), representing another statistically significant difference (*p* < 0.0071 **). Significant differences were also observed for the AA homozygous genotype, this being more frequent in the exposed group than in the unexposed group (*p* < 0.0032 **). Meanwhile, the GG homozygous genotype (wild type) was more frequent in unexposed individuals than in exposed individuals (40% and 0%, respectively), and this difference was also statistically significant (*p* < 0.0001 **) (Table 5 and Figure 7). 

## 3. Discussion

For many years, the carcinogenic effect of certain pesticides on animals and the increased risk in exposed populations of developing malignant tumors have been demonstrated [19]. Indeed, a substantial body of evidence indicates that occupational exposure to pesticides among agricultural workers is linked to a heightened incidence of several diseases, including reproductive disorders, birth defects, cancer, and Alzheimer’s and Parkinson’s diseases [20]. In addition, it has been reported that pesticides induce oxidative damage to DNA, DNA adducts, single- and double-stranded DNA breaks, and chromosomal damage [21]. However, current research has predominantly focused on techniques that primarily reveal the presence of DNA damage resulting from pesticide exposure without providing insight into the type and frequency of CAs or the level of CIN. Understanding these types of damage is crucial for comprehensively assessing the harmful impact of genotoxic agents such as pesticides on chromosomes.

Our results showed a significantly higher frequency of NCAs, CIN, and CH in individuals exposed to pesticides compared to the low frequency observed in unexposed individuals. The findings from our study suggest occupational pesticide exposure has a detrimental impact on chromosomal stability. The average number of CVs and CAs observed in the exposed group was three times higher than that observed in the unexposed group. Furthermore, NCAs were more prevalent and significantly higher in the exposed group.

We also noted a heightened occurrence of statistically significant aneuploidies (gains and/or losses of complete chromosomes) in the exposed group compared to the unexposed group. The observed aneuploidy was unstable, manifesting as variations in chromosome numbers between individual cells within the same individual. Unstable aneuploidy can contribute to CIN and CH by fostering the simultaneous growth of cell subpopulations with differing chromosome numbers [22,23,24,25,26]. In addition to NCAs, we also observed fra and chtb in the same chromosomal region of chromosome 9 (9q12), in 70% and 50% of the exposed individuals, respectively. This observation is particularly significant, as unrepaired fragilities can lead to various chromosomal alterations such as deletions [27], duplications [28], translocations [29], and chromosomal breaks, among others. These alterations have commonly been associated with the initiation and progression of cancer [30,31]. In fact, our results suggest that the fra(9)(q12) could lead to a chromatid break in the same chromosomal region [chtb(9)(q12)]. Indeed, we observed this chromatid break [chtb(9)(q12)] in 50% of exposed individuals. In this regard, it has been suggested that chromatid breaks involve single-stranded and/or double-stranded DNA breaks, which can be induced by reactive oxygen species (ROS). These ROS are highly reactive molecules [32] associated with pesticide exposure [32,33,34]. 

Our results suggest that the presence of fra and chtb in the exposed group could predispose individuals to an increased risk of developing complex chromosomal rearrangements. Complex chromosomal alterations have been associated with the development of several diseases. For instance, chromosomal alterations involving the chromosomal region 9q12 have been observed in acute myeloid leukemia [35], ovary adenocarcinoma [36], diffuse large B-cell lymphoma [37], and multiple myeloma [38], among other diseases. While statistically significant differences were not observed in the frequency of SCA, chtb/chrb, or fra between the exposed and unexposed groups, it is noteworthy that the exposed group exhibited a higher frequency of such alterations.

In our study, the results observed using cytogenetic analysis by GTG banding were consistent with the results obtained using FISH. Specifically, the FISH results revealed that the level of CIN was 18.2 times higher in individuals exposed to pesticides compared to the unexposed group. Additionally, differences in CH were observed, with higher levels in the exposed group than in the unexposed group. These findings suggest a positive correlation between increasing levels of CIN and CH. Indeed, CIN is recognized as a driver of genetic diversity, contributing to CH and thereby enabling cellular adaptation to challenging environments and the development of diseases [39,40]. 

Altogether, our results suggest the detrimental impact of pesticides on chromosomes, marked by a heightened frequency of NCAs, CIN, and CH in the exposed group. This interplay between CIN and CH could contribute to the acquisition of additional chromosomal alterations [41,42] and facilitate cellular adaptation to stressful environments, thereby elevating the risk of disease development [43]. 

Regarding specific chromosomes, we found elevated CIN levels in chromosome 3, this being the chromosome with the highest CIN level among the chromosomes evaluated in the exposed group. The gain in this chromosome has been associated with hormone-secreting pituitary adenoma [44] and with endocrine tumors of the pancreas. Loss of the short arm of this chromosome has been associated with larger tumor size and metastasis in pancreatic cancer [45,46]. Accordingly, chromosome 3 could be an excellent biomarker of CIN since this chromosome was the chromosome that presented the highest levels of CIN in the exposed group.

We observed a positive correlation between age (A), CIN, and TD in the unexposed group. This behavior can be explained by the natural accumulation of chromosomal alterations with age. Indeed, it has been proposed that throughout normal aging, DNA damage occurs continuously on a substantial scale due to various exogenous and endogenous genotoxins [47]. For instance, studies have shown that, on average, up to 105 DNA lesions occur daily in an active mammalian cell and that spontaneous hydrolysis alone is responsible for generating approximately 104 abasic (mostly apurinic) sites [48]. While most of these lesions are effectively repaired, some manage to evade detection, become irreparable, are belatedly repaired, or undergo erroneous repair. Over time, these DNA injuries inevitably accumulate, [49] making genome instability a true hallmark of aging. 

Opposite results were observed in the exposed group since a negative correlation was established between TD and the time of exposure (ET). The observed behavior may be attributed to the potential impact of genotoxic agents on the normal biological processes of individuals who have been exposed to them. The biological mechanisms possibly implicated in the observed response include the following: (i) the toxic response due to exposure to pesticides varying from one organism to another, with this being influenced not only by genetic factors but also by physiological factors (age, diet, nutritional status, hormonal status, state of health, etc.); (ii) the form of metabolization of the pesticides, the route of ingestion, or the affinity between the xenobiotic and the receptor; and (iii) the level of tolerance of cells to genetic damage. All of the above could be determinants for the degree of toxicity and its temporality [50,51]. Extended exposure to pesticides may result in increased DNA damage and subsequently lead to a higher accumulation of NCAs. This accumulation of alterations may surpass the cellular tolerance threshold, triggering apoptosis activation. Consequently, a lower proportion of cells exhibiting CIN is observed. In contrast, with shorter exposure periods to pesticides, cells may tolerate the presence of NCAs, resulting in a higher number of cells with CIN. In fact, oxidative stress (the main effect of pesticides) has been associated with the induction of apoptosis in research conducted on mice [52]. Additionally, a negative correlation between ET and DNA damage has previously been reported in individuals exposed to radiation [53]. 

The deleterious effect of pesticides on chromosomes could be caused by the direct interaction of pesticides with DNA or by the oxidative stress generated by such exposure [54,55,56,57,58]. Furthermore, DNA repair mechanisms could also be connected to the high frequency of CAs and CIN we observed in the exposed group. Indeed, several DNA repair mechanisms respond to this damage and help maintain cell integrity, so alteration of such mechanisms could modulate the individual’s susceptibility to DNA repair and the development of various diseases. In fact, the ability to repair DNA damage and metabolize environmental pesticide by-products is genetically determined. Individuals with deficiencies in genes associated with DNA repair mechanisms may experience elevated levels of irreversible genetic damage even with low-intensity exposure. The presence of such deficiencies could indicate susceptibility to exposure [10]. Indeed, interindividual differences contribute to variations in susceptibility and response to different pesticide exposures, potentially heightening health risks. We studied such interindividual differences in the exposed and unexposed groups by evaluating polymorphic variants in genes that code for glutathione S transferases (*GST*) and for DNA damage repair (*XRCC1*) [13].

In this regard, we found that polymorphic variants at exon 5 (AG) in the *GSTP1* gene and at exon 10 (GA) in the *XRCC1* gene were more frequent (and statistically significant) in the exposed group than in the unexposed group. The results of our study suggest that CIN was higher in pesticide-exposed individuals carrying the heterozygous *GSTP1* Ile-Val genotype than in those with the other two genotypes (Ile-Ile and Val-Val), as well as in pesticide-exposed individuals carrying the heterozygous *XRCC1* (exon 10) Arg-Gln genotype compared to the other two genotypes (Arg-Arg and Gln-Gln). Our findings align with studies that have correlated the heterozygous *GSTP1* Ile-Val genotype with an enzyme deficiency in xenobiotic metabolism, leading to the accumulation of active xenobiotic-derived metabolites, potential chromosomal damage, and a lower likelihood of survival in certain types of cancer, such as esophageal cancer [59]. In fact, recent studies have highlighted that individuals harboring polymorphic variants at exon 5 (AG) and carrying the heterozygous *GSTP1* Ile-Val genotype exhibit diminished enzymatic activity. This renders them more vulnerable to oxidative stress and impairs their ability to detoxify carcinogenic xenobiotics [17].

Furthermore, a recent study indicated that the heterozygous *GSTP1* Ile-Val genotype was the most common in a Brazilian population exposed to mercury. This polymorphism was linked to a potential abnormal somatosensory signal and neuropathy [60]. Additionally, for the homozygous *GSTP1* Val-Val genotype, associations were reported suggesting a protective effect against mercury accumulation [60]. This is consistent with our findings, as the *GSTP1* Val-Val genotype showed the highest frequency (60%) in the unexposed group. Additional studies have reported associations between the *GSTP1* Val-Val genotype and improved clinical outcomes in patients with breast, colon, or multiple myeloma cancer following chemotherapy [61]. 

Regarding the assessment of polymorphisms at exon 6 in the *XRCC1* gene, we observed a predominant presence of the wild-type CC homozygous genotype in exposed and unexposed individuals. This observation is consistent with findings from previous studies [62]. Concerning polymorphisms at exon 10 of the *XRCC1* gene, we observed a higher prevalence of the heterozygous genotype GA in the exposed group in comparison to the control group. This observation is significant because prior studies have suggested that the presence of this polymorphism is associated with diminished repair capacity, increased mutagenic sensitivity, and elevated levels of DNA damage [17]. Similarly, we observed a predominance of the GG genotype in unexposed individuals, consistent with previous findings [63].

Genetic polymorphisms in genes related to pesticide metabolism represent potential candidates that could impact susceptibility to pesticide-induced toxicity. These can be attributed to the fact that proteins encoded by different genotypes have the potential to influence the biotransformation of substrates [10]. Indeed, the relevance of genetic polymorphisms as modifiers of human disease has gained considerable attention in the past decade. For instance, polymorphism in the coding regions of the *GSTP1* gene may affect the enzymatic activity of pesticide-metabolizing enzymes and could potentially contribute to increased toxicity associated with chronic pesticide exposure [64,65,66]. Certainly, the presence of non-functional GSTs due to polymorphisms has been linked to an elevated risk of DNA damage and the development of cancer, especially in the context of occupational exposure to pesticides. In fact, it has been indicated that the Ile105Val polymorphism of *GSTP1* exhibits the highest expression in lung tissue and is associated with several cancer types [67]. 

Overall, our findings suggest a potential association between polymorphism at exon 5 (AG) in the *GSTP1* gene and at exon 10 (GA) in the *XRCC1* gene and the elevated levels of CIN and CH in individuals exposed to pesticides. Therefore, our findings may enhance the understanding of the potential adverse effects associated with pesticide exposure, which could potentially contribute to the development of various diseases, including cancer. Indeed, recent research highlights how the interaction between genetic polymorphisms and pesticide exposure significantly influences the likelihood of developing various diseases [10]. For instance, while polymorphic variants in the *GSTP1* gene have been associated with the metabolism of pesticides [68,69,70] and with the development of neoplasms [71], polymorphisms in the *XRCC1* gene have been linked to an elevated risk of DNA damage caused by pesticide exposure [72]. Polymorphisms in the *XRCC1* gene may lead to a reduction in DNA repair capacity caused by increased oxidative stress in individuals exposed to pesticides. 

## 4. Materials and Methods

### 4.1. Study Groups

The study was carried out on a group of ten (10) individuals from the town of Aquitania, Colombia, farmers routinely “exposed” to pesticides (exposed group), and ten (10) individuals without indication of previous occupational exposure to pesticides (unexposed group). Each subject included in the study was interviewed in order to record possible confounding factors such as diseases, age, smoking and drinking habits, exposure time to pesticides (in exposed individuals), frequency of exposure to pesticides, type of pesticide mixture, and dispersion mechanisms. Both exposed and unexposed individuals who had cancer or had received radiation therapy, chemotherapy, or other prolonged medical treatment were excluded from the study. 

The study was conducted in accordance with the declaration of Helsinki and approved by the ethics committee of Universidad Pedagógica y Tecnológica de Colombia (date of approval 4 June 2021). Written informed consent was obtained from each study participant.

### 4.2. Blood Sample Collection

Ten (10) milliliters of peripheral blood were collected from exposed and unexposed individuals by venous puncture in two vacutainer plastic tubes: one tube with heparin (5 mL) and the other with EDTA (5 mL). Standardized harvest protocols were applied. 

### 4.3. Cytogenetic Assays

Metaphases were obtained using standard harvesting protocols for GTG banding and molecular cytogenetic analysis. Briefly, 1 mL of heparinized peripheral blood was cultured in duplicates in 5 mL of RPMI-1640 medium (Sigma, St. Louis, MO, USA), supplemented with 150 µL of phytohemagglutinin-M (Gibco, Life Technologies, Waltham, MA, USA) and 10% fetal bovine serum (FBS) (Sigma, St. Louis, MO, USA). The cultures were incubated for 72 h at 37 °C in a 5% CO_2_ atmosphere. After 72 h, N-Deacetyl-N-methyl colchicine solution (0.0001 g/mL final conc.) (Sigma, St. Louis, MO, USA) was added to cultures 25 min before cell harvesting. Then, cells were treated with hypotonic solution (KCl solution) at a concentration of 0.075 M, fixed with Carnoy’s fixative (3:1 methanol to acetic acid) three times, and spread on glass slides. Finally, the chromosomal preparations were banded with GTG banding using trypsin solution (0.25%) (Gibco, Life Technologies, Waltham, MA, USA) and Giemsa stain (Sigma, St. Louis, MO, USA). Image acquisition and karyotyping of metaphases was performed using an Olympus microscope with the cytogenetic software Cytovision system 7.4 (Leica Biosystems, Richmond, IL, USA). Characterization of numerical and structural chromosomal alterations and chromosomal variants [1qh+, 9qh+, fra, chrb, and chrb], were evaluated on a total of 731 metaphases. CAs and CVs were described according to the International System for Human Cytogenomic Nomenclature (ISCN) 2020 [73].

### 4.4. Fluorescence In Situ Hybridization (FISH) Assays

In order to evaluate CIN and CH on previously obtained interphase nuclei spreads, we performed FISH by applying five (5) centromeric probes (CEP) labeled with different fluorochromes. The probes used included probes for chromosomes 2 and 3 (orange fluorochrome), 11 and 15 (green fluorochrome) (all from Cytocell, Cambridge), and for chromosome 17 (green fluorochrome) (Vysis. Abbott, Downers Grove, IL, USA). Dual-color FISH was performed on the interphase nuclei spreads for CEP2 and CEP11 and for CEP3 and CEP15. CEP17 was evaluated in a single assay.

Specifically, FISH was performed as follows: the interphase nuclei spreads were dehydrated for one minute in ethanol at different concentrations (70%, 85%, 90%, and 100%). After dehydration, the mixture of probes corresponding to each assay was added to the interphase nuclei spreads, and they were subsequently denatured at 75 °C for 2 min and hybridized overnight at 37 °C using the Top Brite system (Resnova, Roma, Italy). At the end of the hybridization time, the interphase nuclei spreads were subjected to astringency washes, dehydrated in ethanol series, and colored with 4′,6-diamidino-2-phenylindole (DAPI) (Cytocell, Cambridge, United Kingdom ). Thus obtained, the interphase nuclei spreads from each individual were analyzed and processed by reading at least ten randomly selected areas with an Olympus microscope and Cytovision system 7.4 cytogenetic software.

### 4.5. CIN and CH Evaluation

CIN was evaluated for each chromosome in 100 separate and well-defined nuclei. The CIN level for each exposed and unexposed individual was determined in two steps. The first step consisted of determining the CIN level for each of the five chromosomes separately, with the CIN level corresponding to the percentage of nuclei with a CEP signal number different from the modal number (most frequent number of chromosomes in a cell population). The second step consisted of determining the average percentage of CIN of the five chromosomes analyzed [74,75]. According to the level of CIN, each exposed and unexposed individual was classified as having low CIN (CIN < 25%) or high CIN (CIN ≥ 25%) [76,77]. The CIN levels observed in each of the exposed individuals were compared with the CIN levels observed in the control group (unexposed). 

The presence of different cell populations with different levels of aneuploidy in the same individual (CH) was calculated for chromosomes 2, 3, 11, 15, and 17 in each exposed and unexposed individual with the true diversity index (TD). TD integrates both the number and abundance of different cell populations within each cell [78,79,80]. According to the level of CH, each exposed and unexposed individual was classified as having low (<1.5), intermediate (CH > 1.62 < 2), or high CH (CH > 2).

### 4.6. Polymerase Chain Reaction—Restriction Fragment Length Polymorphism (PCR-RFLP) 

DNA extraction was performed using the salting out method, with a commercial DNA extraction from blood kit (Qiagen, Valencia, CA, USA), according to the manufacturer’s instructions. Briefly, 5 mL of blood was treated with red blood cell lysis buffer, white blood cell lysis buffer, proteinase K, and protein precipitant solution. DNA was precipitated using cold isopropanol, resuspended in sterile water, and then quantitated by measuring OD260. DNA integrity was evaluated on the basis of sharp intact bands using gel electrophoresis. The *GSTP1* and *XRCC1* polymorphic sites were investigated using the PCR—RFLP technique. Two primers (forward and reverse) were used to determine the genotype and allele status of *GSTP1* gene exon 5, while four primers were used to determine the genotype and allele status of *XRCC1* gene exons 6 and 10 (Table 6). 

The PCR for the *GSTP1* (exon 5) and *XRCC1* (exons 6 and 10) genes was carried out in a final reaction of 10 μL with 2.5 μL of H_2_O, 6.25 μL of master Mix (Taq, MgCl_2_, buffer, dNTP) (Promega), 1.25 μL of each primer, and 2 μL of DNA (50 ng/μL). The PCR cycle started at 95 °C for 5 min and was followed by 35 cycles at 95 °C for 30 s (s), at 62 °C for 30 s, at 72 °C for 45 s, and finally at 72 °C for 5 min to allow a full extension of all PCR fragments. 

The PCR products for the Ile to Val substitution in *GSTP1* gene exon 5 were digested with BsmA1 for 24 h at 37 °C and then electrophoresed on 2.5% agarose. A single fragment of 176 bp corresponded to the AA genotype, while the presence of two fragments of 93 and 83 bp corresponded to the homozygous GG genotype. Heterozygous genotypes (AG) contained all three fragments (176 bp, 93 bp, and 83 bp). 

The PCR products for the Arg to Trp substitution in *XRCC1* exon 6 were digested with PvuII for 24 h at 37 °C and then electrophoresed on 2.5% agarose. The products were identified by gel electrophoresis using 2.5% agarose. A fragment of 485 bp corresponded to the CC genotype, while the presence of two fragments of 396 and 89 bp, corresponded to the TT genotype. Heterozygous genotypes (CT) contained all three fragments (485 bp, 396 bp and 89 bp). 

The PCR products for the Arg to Gln substitution in *XRCC1* exon 10 were digested with MspI for 24 h at 37 °C and then electrophoresed on 2.5% agarose. The presence of a fragment of 242 bp corresponded to the GG genotype, while the presence of two fragments of 148 and 94 bp corresponded to the AA genotype. Heterozygous genotypes (GA) contain all three fragments (242 bp, 148 bp, and 94 bp).

### 4.7. Statistical Analysis

Fisher’s exact test, Student’s *t*-test, and the Wilcoxon test were performed to compare the data of CAs, CVs, CIN, and TD with a parametric and non-parametric distribution. The Kruskal–Wallis test was employed for data with a non-parametric distribution to compare CIN and TD among the analyzed chromosomes in this study. Homogeneity and data normality of variances were evaluated using Bartlett’s test and the Shapiro–Wilk test, respectively. A multivariate analysis utilizing the Spearman correlation coefficient was conducted to assess potential associations between CAs, CVs, levels of CIN and CH, and variables such as age and duration of pesticide exposure within both the exposed and unexposed groups.

The genotype frequencies of *GSTP1* and *XRCC1* gene polymorphisms were compared using Fisher’s exact test. The Hardy–Weinberg equilibrium was evaluated for each polymorphism using the chi-square test for the exposed and unexposed individuals separately. Data from exposed and unexposed individuals were compared. Statistical analyses were conducted using R Studio version 4.0.2, with *p*-values < 0.05 being considered statistically significant (* *p* ≤ 0.05, ** *p* ≤ 0.01, and *** *p* ≤ 0.001). CIN and TD are expressed as mean ± SD.

## 5. Conclusions

Despite being derived from a limited sample size, our results suggest chromosomal damage resulting from pesticide exposure. The genotoxicity observed in this study due to pesticide exposure could be considered as an early indicator for the potential development of diseases. The accumulation of numerical chromosomal alterations is a crucial step for the onset of many types of diseases, including cancer. Furthermore, our results suggest that individuals carrying polymorphic variants at exon 5 (AG) in the *GSTP1* gene and at exon 10 (GA) in the *XRCC1* gene could face an elevated risk of DNA damage induced by pesticide exposure. However, it is important to emphasize the necessity of validating our findings across a larger number of individuals. Our findings underscore the importance of educating exposed farmers about the potential adverse effects of pesticides. Furthermore, they emphasize the pivotal role that relevant authorities must assume in guaranteeing the implementation of protective measures for farmers working in agricultural fields.

## Figures and Tables

**Table 1:** General characteristics of the exposed and unexposed groups.

	Exposed	Unexposed
Number	10	10
Age (mean ± SD)	39.20 ± 17.78	39.00 ± 17.44
Sex (n)		
Male	3	3
Female	7	7
Exposure months (mean ± SD)	180.2 ± 239.9	0
Tobacco smoking (n)		
Smokers	0	0
Non-smokers	10	10
Alcohol consumption (n)		
Alcohol consumers (1/week)	2	1
Alcohol consumers (sporadic)	7	8
Non-alcohol consumers	1	1

**Table 2:** Characteristics of the exposed group.

Characteristic	Number
Type of exposure	
Dermal	7
Respiratory	3
Method of irrigation	
Machine	7
Bomb	2
Mixed	1
Frequency of irrigation	
2 times a month	4
1 time a week	4
Unknown	2

**Table 3:** Frequency of chromosomal alterations and chromosomal variants identified in the exposed and unexposed groups.

CAs and CVs	Number of Alterations		
	E	UE	*p*+
	n (%)	n (%)	
NCAs	151 (43.0)	33 (8.7)	0.0001 **
Monosomies	42 (12.0)	21 (5.5)	0.2159
Trisomies	29 (8.3)	2 (0.5)	0.0349 *
mar	38 (10.8)	4 (1.1)	0.0050 **
end	21 (6.0)	2 (0.5)	0.1184
Polyploidies	20 (5.7)	3 (0.8)	0.1184
SCAs	20 (5.7)	15 (3.9)	0.7475
chtb/chrb	20 (5.7)	7 (1.8)	0.2790
fra	42 (12.0)	22 (5.8)	0.2159
fra(9)(q12)	29 (8.3)	16 (4.2)	0.3727
9qh+	4 (1.1)	5 (1.3)	1
Total alterations	237	82	
Mean	47.4	16.4	
SD	59.4	11.5	
*p*++	0.125		

Abbreviations: E—exposed farmer; UE—unexposed control; n—frequency; CVs—chromosomal variants; CAs—chromosomal alterations; NCAs—numerical chromosomal alterations; mar—marker chromosome; end—endoreduplication; SCAs—structural chromosomal alterations; chtb/chrb—chromatid break/chromosomal break; fra—fragilities; fra(9)(q12)—fragility on the long arm of chromosome 9, region 1, band 2; 9qh+, heterochromatin increased on the long arm of chromosome 9; SD—standard deviation. Notes: * Statistically significant difference relative to the unexposed group at *p* ≤ 0.05. ** Statistically significant difference relative to the unexposed group at *p* ≤ 0.01 (*p*+: Fisher’s exact test; *p*++: Wilcoxon signed rank test).

**Table 4:** Frequency of chromosomal alterations and chromosome variants identified in paired exposed and unexposed individuals.

CVs and CAs	Number of Individuals		
	E	UE	*p*
	n (%)	n (%)	
NCAs	10 (100)	8 (80)	0.0001 **
Monosomies	10 (100)	8 (80)	0.0001 **
Trisomies	10 (100)	3 (30)	0.0001 **
mar	9 (90)	3 (30)	0.0001 **
end	5 (50)	1 (10)	0.0001 **
Polyploidies	6 (60)	2 (20)	0.0001 **
SCAs	9 (90)	7 (70)	0.0007 **
chtb/chrb	6 (60)	4 (40)	0.0071 **
fra	8 (80)	7 (70)	0.1412
9qh+	2 (20)	2 (20)	1
Total	10	10	

Abbreviations: E—exposed farmer; UE—unexposed control; n—frequency; CVs—chromosomal variants; CAs—chromosomal alterations; NCAs—numerical chromosomal alterations; mar—marker chromosome; end—endoreduplication; SCAs—structural chromosomal alterations; chtb/chrb—chromatid break/chromosomal break; fra—fragilities; 9qh+—heterochromatin increased in the long arm of chromosome 9. Notes: ** Statistically significant difference relative to the unexposed group at *p* ≤ 0.01 (Fisher’s exact test).

**Table 5:** SNP polymorphisms in exposed and unexposed individuals.

Gene	SNP Genotype	Exposed	Unexposed	*p*
*GSTP1* Exon 5	Ile105Val (A→G)			
	AA	1 (10%)	2 (20%)	0.0734
	AG	7 (70%)	2 (20%)	0.0001 **
	GG	2 (20%)	6 (60%)	0.0001 **
*XRCC1* Exon 6	Arg194Trp (C→T)			
	CC	8 (80%)	8 (80%)	1
	CT	2 (20%)	2 (20%)	1
	TT	0	0	1
*XRCC1* Exon 10	Arg399Gln (G→A)			
	GG	0	4 (40%)	0.0001 **
	GA	6 (60%)	4 (40%)	0.0071 **
	AA	4 (40%)	2 (20%)	0.0032 **

Abbreviations: SNP—single-nucleotide polymorphism. Notes: ** Statistically significant difference relative to the unexposed group at *p* ≤ 0.01 (Fisher’s exact test).

**Table 6:** Primer sequences selected on the *GSTP1* and *XRCC1* genes.

Gene	Primer	Primer Sequences	Exon	PCR Product	Restriction Enzyme
*GSTP1*	Ile105Val (A→G)	F: 5′ACCCCAGGGCTCTATGGGAA3′	5	176 bp	BsmA1
		R: 5′TGAGGGCACAAGAAGCCCCT3′			
*XRCC1*	Arg194Trp (C→T)	F: 5′GCCAGGGCCCCTCCTTCAA3′	6	485 bp	Pvu II
		R: 5′TACCCTCAGACCCACGAGT3′			
	Arg399Gln (G→A)	F: 5′CCCCAAGTACAGCCAGGTC3′	10	242 bp	MspI
		R: 5′TGTCCCGCTCCTCTCAGTAG3′			

## Data Availability

The original contributions presented in the study are included in the article/Supplementary Materials. Further inquiries can be directed to the corresponding authors.

## Supplementary Materials

The following supporting information can be downloaded at https://www.mdpi.com/article/10.3390/ijms25084167/s1.
