# Supplementary material for: Chromosomal Damage, Chromosome Instability, and Polymorphisms in GSTP1 and XRCC1 as Biomarkers of Effect and Susceptibility in Farmers Exposed to Pesticides
Source: Int J Mol Sci. 2024 Apr 10;25(8):4167. doi: 10.3390/ijms25084167 (PMC11050655; doi:10.3390/ijms25084167)
Supplement: Supplementary file 1 [file ijms-25-04167-s001.zip › Supplementary Tables.pdf]

Supplementary Material

Table S1. Detailed characteristics of the groups studied

E	Age (y)	Exposure (m)	Habits		UE	Age (y)	Exposure (m)	Habits	
			S	D				S	D
1	48	24	NS	SP	1	47	-	NS	SP
2	40	24	NS	SP	2	41	-	NS	W
3	70	780	NS	W	3	68	-	NS	SP
4	30	108	NS	SP	4	29	-	NS	ND
5	22	60	NS	SP	5	22	-	NS	SP
6	24	36	NS	W	6	24	-	NS	SP
7	19	50	NS	SP	7	19	-	NS	SP
8	60	384	NS	SP	8	60	-	NS	SP
9	26	96	NS	SP	9	24	-	NS	SP
10	53	240	NS	ND	10	54	-	NS	SP
**Mean**	39.20	180.2			**Mean**	39.00			
**Median**	35.00	78.00			**Median**	35.00			
**SD**	17.78	239.9			**SD**	17.44			

Abbreviations: E, Exposed; UE, unexposed; S, Smoking; NS, Non-smoker; D, Drinking; ND, Non-drinker; SP, sporadic; W, week; m, month; y, year; SD, Standard Deviation

Table S2. Chromosomal Instability (CIN) and True Diversity Index (TD) for Exposed (E) and Unexposed (UE) groups

E	CIN						TD					
	CEP2	CEP3	CEP11	CEP15	CEP17	Mean	CEP2	CEP3	CEP11	CEP15	CEP17	Mean
1	10%	47%	12%	26%	78%	35%	1,48	3,06	1,52	2,03	7,1	3,04
2	48%	49%	54%	40%	58%	50%	3,26	3,12	2,82	2,56	3,55	3,06
3	10%	52%	30%	27%	10%	26%	1,48	3,27	1,84	2,15	1,47	2,04
4	44%	50%	31%	25%	74%	45%	3,44	3,34	2,37	2,07	6,01	3,45
5	42%	38%	41%	36%	40%	39%	2,82	2,96	2,47	2,36	3,37	2,79
6	35%	44%	51%	37%	31%	40%	2,77	3,14	2,31	2,31	2,48	2,61
7	34%	35%	51%	34%	29%	37%	2,77	2,53	2,18	2,45	2,38	2,46
8	23%	48%	45%	37%	27%	36%	2,02	3,29	2,37	2,19	2,42	2,46
9	27%	46%	49%	21%	19%	32%	2,28	2,87	2,23	1,95	1,91	2,25
10	29%	60%	51%	30%	27%	39%	2,59	3,53	2,31	2,23	2,36	2,60
**UE**	**CIN**						**TD**					
	**CEP2**	**CEP3**	**CEP11**	**CEP15**	**CEP17**	**Mean**	**CEP2**	**CEP3**	**CEP11**	**CEP15**	**CEP17**	**Mean**
1	18%	49%	21%	22%	15%	25%	1,81	3,29	1,92	2,0	1,58	2,12
2	17%	16%	20%	18%	31%	20%	1,67	1,67	1,76	1,71	2,45	1,85
3	18%	18%	19%	31%	21%	21%	1,87	1,81	1,89	2,09	1,8	1,89
4	13%	18%	18%	19%	27%	19%	1,65	1,81	1,71	1,73	2,32	1,84
5	17%	20%	30%	15%	24%	21%	1,82	1,98	1,98	1,58	1,97	1,87
6	16%	16%	22%	24%	26%	21%	1,78	1,78	1,84	1,89	1,98	1,85
7	8%	24%	15%	21%	17%	17%	1,39	2,06	1,52	1,98	1,82	1,75
8	9%	25%	18%	26%	30%	22%	1,43	1,97	1,61	2,04	2,83	1,97
9	9%	13%	11%	18%	22%	15%	1,47	1,64	1,48	1,66	2,06	1,66
10	10%	18%	12%	9%	32%	16%	1,48	1,66	1,52	1,35	2,15	1,63

Abbreviations: E, Exposed individuals; UE, Unexposed individuals; CEP2, centromeric probe for chromosome 2; CEP3, centromeric probe for chromosome 3; CEP11, centromeric probe for chromosome 11; CEP15, centromeric probe for chromosome 15; CEP17, centromeric probe for chromosome 17; CIN, Chromosomal Instability; TD, True Diversity index

SUPPLEMENTARY FIGURE LEGEND

Figure S1. Multivariate analysis with Pearson correlation coefficient between clonal heterogeneity (CH) and clinicopathologic characteristics. Values greater than 0.5 are indicative of a statistically significant correlation. No correlation was found between CH with any of the variables studied: histotype (HT), tumor size (T), lymph nodes (N), lymphovascular invasion (LI), progesterone receptor (PR), HER2 and KI67.
