# Supplementary figures and images for: Chromosomal Damage, Chromosome Instability, and Polymorphisms in GSTP1 and XRCC1 as Biomarkers of Effect and Susceptibility in Farmers Exposed to Pesticides
Source: Int J Mol Sci. 2024 Apr 10;25(8):4167. doi: 10.3390/ijms25084167 (PMC11050655; doi:10.3390/ijms25084167)

A

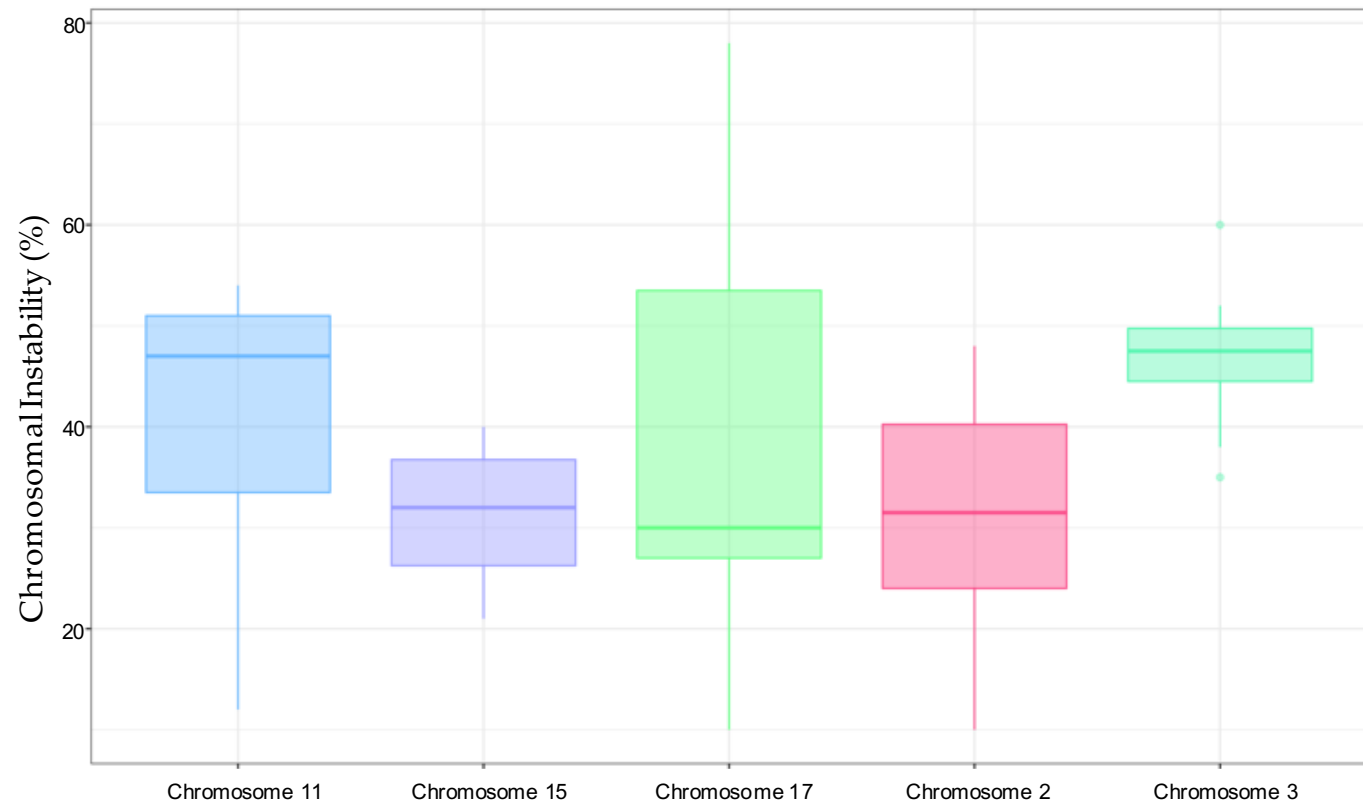

B

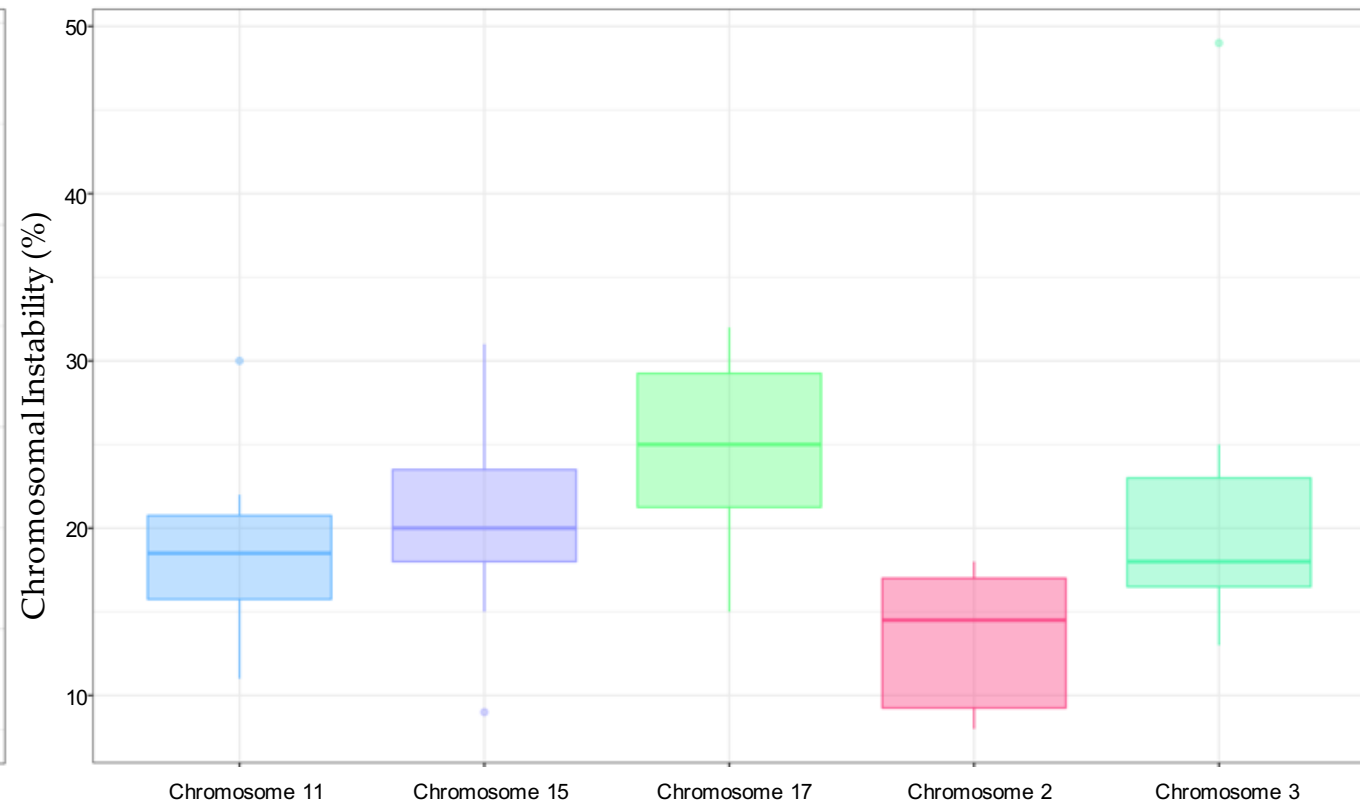

Supplement: Supplementary file 1 [file ijms-25-04167-s001.zip › Supplementary Figure S1.pdf]
